# Mechanism of tumor rejection with doublets of CTLA-4, PD-1/PD-L1, or IDO blockade involves restored IL-2 production and proliferation of CD8^+^ T cells directly within the tumor microenvironment

**DOI:** 10.1186/2051-1426-2-3

**Published:** 2014-02-18

**Authors:** Stefani Spranger, Holly K Koblish, Brendan Horton, Peggy A Scherle, Robert Newton, Thomas F Gajewski

**Affiliations:** 1 Department of Pathology, University of Chicago, 5841 S. Maryland Ave, Chicago, IL 60637, USA; 2 Department of Medicine, University of Chicago, Chicago, IL 60637, USA; 3 Incyte Corporation, Wilmington, DE 19880, USA

**Keywords:** Anti-CLTA-4, PD-1/PD-L1, IDO inhibitor, Combinatorial immunotherapy, Tumor-infiltrating lymphocytes, T cell anergy/exhaustion, Tumor microenvironment, Immune inhibitory pathways

## Abstract

**Background:**

Blockade of immune inhibitory pathways is emerging as an important therapeutic modality for the treatment of cancer. Single agent treatments have partial anti-tumor activity in preclinical models and in human cancer patients. Inasmuch as the tumor microenvironment shows evidence of multiple immune inhibitory mechanisms present concurrently, it has been reasoned that combination therapies may be required for optimal therapeutic effect.

**Methods:**

To test this notion, we utilized permutations of anti-CTLA-4 mAb, anti-PD-L1 mAb, and/or the IDO inhibitor INCB23843 in the murine B16.SIY melanoma model.

**Results:**

All three combinations showed markedly improved tumor control over single treatments, with many mice achieving complete tumor rejection. This effect was seen in the absence of vaccination or adoptive T cell therapy. The mechanism of synergy was investigated to examine the priming versus effector phase of the anti-tumor immune response. Only a minimal increase in priming of anti-tumor T cells was observed at early time points in the tumor-draining lymph nodes (TdLN). In contrast, as early as three days after therapy initiation, a marked increase in the capacity of tumor-infiltrating CD8^+^ T cells to produce IL-2 and to proliferate was found in all groups treated with the effective combinations. Treatment of mice with FTY720 to block new T cell trafficking from secondary lymphoid structures still enabled restoration of IL-2 production and proliferation by intratumoral T cells, and also retained most of the tumor growth control.

**Conclusions:**

Our data suggest that the therapeutic effect of these immunotherapies was mainly mediated through direct reactivation of T cells in situ. These three combinations are attractive to pursue clinically, and the ability of intratumoral CD8^+^ T cells to produce IL-2 and to proliferate could be an important biomarker to integrate into clinical studies.

## Background

Despite expression of numerous antigens, tumor evasion from host immunity still occurs. Over the past several years, a working model has emerged suggesting the existence of at least two major categories of immune resistance based on biologic features of the tumor microenvironment [1]. One major subset shows infiltration with CD8^+^ T cells at baseline, along with a specific chemokine profile and a type I interferon (IFN) transcriptional signature, all indicative of active Th1-type inflammation. These tumors appear to resist the ongoing immune response through the dominant inhibitory effect of immune suppressive mechanisms. In contrast, the other major subset lacks these chemokines and T cell markers and appears to escape immune effects through immunologic ignorance or exclusion. With this as a working model, one might envision distinct immunotherapies being necessary for optimal therapeutic effects in these two patient groups. This conceptual framework is being integrated into biomarker development as novel immunotherapies are being explored in patients [2]. While most of these analyses have been carried out on biopsy material from patients with advanced melanoma, similar results are emerging in other solid tumors, including lung cancer, ovarian cancer, colorectal cancer, breast cancer, and head and neck cancer [3-6].

Focusing in on the T cell “inflamed” tumor subset, at least four immune inhibitory mechanisms have been identified to be involved in human specimens and validated mechanistically in preclinical models. These are expression of the ligand PD-L1 (programmed death-ligand 1), which can engage the inhibitory receptor PD-1 (programmed death-receptor 1) on activated T cells; presence of the tryptophan-catabolizing enzyme indoleamine-2,3-dioxygenase (IDO), which exploits the exquisite sensitivity of T cells to tryptophan depletion; infiltration with FoxP3^+^ regulatory T cells (Tregs), which can mediate extrinsic suppression of effector T cell function; and T cell-intrinsic anergy, characterized by defective IL-2 (interleukin-2) production and proliferation and driven in part through the transcription factor Egr2 (Early growth response protein 2) [6-11]. We recently have observed that increased expression of PD-L1, IDO, and FoxP3^+^ Tregs in the melanoma tumor microenvironment is driven by infiltrating CD8^+^ T cells, arguing that these mechanisms are part of an immune-intrinsic negative feedback loop [12]. Thus, immunotherapies aiming to uncouple these pathways may be most effective in tumors showing the T cell-infiltrated phenotype, during the negative feedback phase of a chronic, smoldering immune response.

The first successful immunotherapy aiming to block a negative regulatory pathway on T cells is the anti-CTLA-4 (Cytotoxic T-Lymphocyte Antigen-4) mAb ipilimumab, which was approved by the FDA (Food and Drug Administration) in 2011 for treatment of patients with advanced melanoma [13]. CTLA-4 is an inhibitory receptor also expressed by tumor-infiltrating T cells. This success has catalyzed extensive investigation into the potential for inhibitors of other immune suppressive pathways to have clinical activity in advanced cancer patients. Early clinical results have been reported with anti-PD-1 and anti-PD-L1 mAbs [14], IDO inhibitors [15], and Treg depletion targeting surface CD25 [16,17]. Numerous additional immunotherapeutic agents are being developed that target a plethora of positive or negative immunoregulatory molecules. These include agonistic Abs against 4-1BB, Ox40, ICOS (Inducible T-cell COStimulator), and CD40; blocking Abs against LAG3 (Lymphocyte-activation gene 3), B7-H3, B7-H4, Tim3, and KIRs (killer inhibitory receptors); and the cytokines IL-7, IL-15, and IL-21 [18-26], among others. Based on the presumption that even the best of these agents will have modest single agent activity, and because of the complexity of immune regulation in vivo that involves networks of regulatory pathways, it is envisioned that combination immunotherapies ultimately will be required for maximal therapeutic benefit.

The prospect of investigating combination immunotherapy doublets in early phase clinical trials in an empiric fashion without solid mechanistic data seems daunting and perhaps impossible to pursue based on available patient resources. We recently have assembled a framework for logical combination of immunotherapeutic agents based on presumed modes of action [27]. This framework provides a motivation for testing such logical combinations in suitable mouse preclinical models to aid in the prioritization of regimens for clinical development. Mouse models also facilitate elucidation of the mechanism by which two interventions may be synergistic.

In the current report, we have started with αCTLA-4 mAb as a successful backbone, along with αPD-L1 and IDO inhibition. As a tumor model, we utilized B16 melanoma, which does develop an “inflamed” tumor microenvironment at early time points that leads to accumulation of PD-L1, IDO, and Tregs over time [12]. We found that doublets of αCTLA-4 +/− αPD-L1 +/− an IDO inhibitor each showed improved tumor control in vivo. Mechanistic studies revealed that the major biologic effect of the successful doublets was restoration of IL-2 production and proliferation by CD8^+^ T cells within the tumor microenvironment. Our results provide support for continued development of these combinations in patients, and also suggest a new predictive biomarker that should be integrated into clinical development.

## Results

### Combinatorial blockade of CTLA-4, PD-L1 or IDO pathways results in improved tumor control in vivo

**Figure 1:**
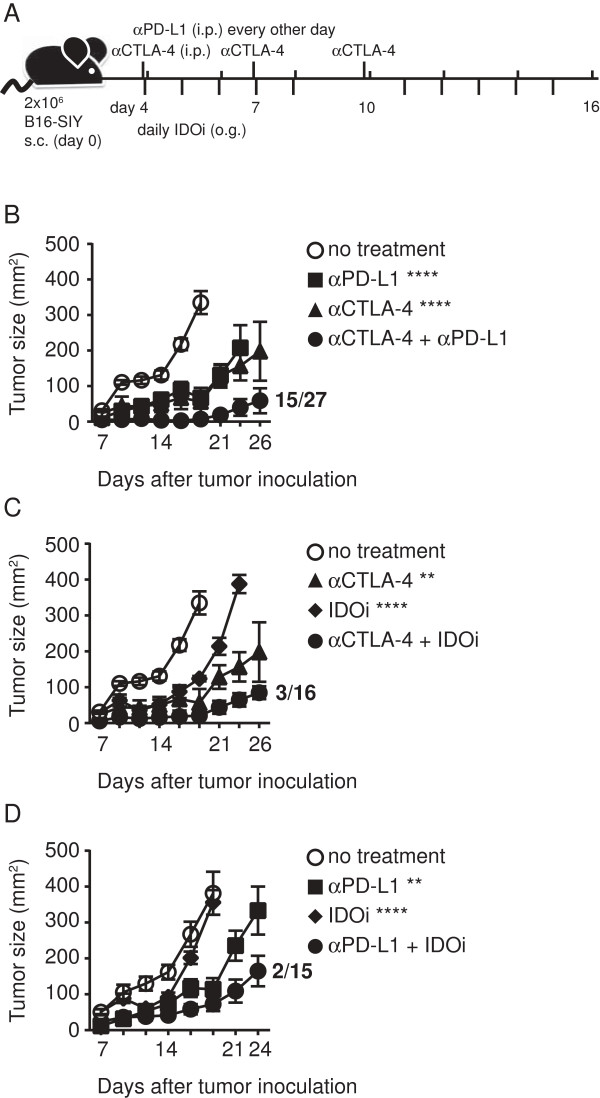
**Pairwise combinations of αCTLA-4, αPD-L1 or IDOi blockade results in retarded tumor outgrowth. A**. Schema of experimental design illustrating the time points of drug administration throughout the experiment. αCTLA-4 was administered on day 4, 7, 10 i.p., αPD-L1 on days 4, 6, 8, 10, 12, 14, 16 i.p. and IDOi was given on a 5 day/2 day off regimen via oral gavage Monday-Friday. **B**-**D**. Tumor outgrowth measured in mm^2^ comparing the single treatment to the respective combined double treatment of **B**. αCTLA-4 and αPD-L1 C. αCTLA-4 and IDOi and **D**. αPD-L1 and IDOi (pool of 3 experiments). Depicted are means +/− SEM of 6 mice from one representative experiment. All experiments were at least done three times with the same overall result. Numbers next to curves indicate # of survivors/total number of mice for all experiments done. Significance to the single treatments was tested using a two-way-Anova with Bonferroni post-test and is shown in the figure while all treatments regimens were significantly different to the no treatment control (**** <0.0001, ** <0.01).

Single blockade of the immune-inhibitory pathways CTLA-4, PD-1/PD-L1, or IDO has been shown to have modest yet significant impact on tumor growth kinetics and to improve tumor-specific immune responses in various mouse models in vivo [28-33]. With the notion that combinatorial blockade might result in additive or synergistic effects, we pursued the following experimental treatment strategies (illustrated in Figure 1A). On day 0, B16.SIY cells were inoculated subcutaneously in the flank of C57BL/6 mice. After allowing the tumor cells to engraft, therapy was initiated on day 4. The αCTLA-4 mAb (clone: UC10-4F10-11) was given at three single time points [34]. The αPD-L1 mAb (clone: 10F.9G2) was given every other day throughout the treatment protocol, and the IDO inhibitor (INCB23843) was given daily Monday-Friday via oral gavage [35]. Treatment with single agents versus doublet combinations was compared, using tumor growth measured twice per week as the endpoint. For all three double treatments, we observed improved tumor control compared to the corresponding single regimens. In particular, the combination of αCTLA-4 and αPD-L1 resulted in 15 complete responders out of a total of 27 treated mice (55.5%; Figure 1B). Lower numbers of complete responders were found for the combination of αCTLA-4 and IDOi (3/16; Figure 1C) and αPD-L1 and IDOi (2/15; Figure 1D) (complete responders see Additional file 1: Table S1). In addition to the tested doublet therapies we also conducted experiments using the triple combination of αCTLA-4, αPD-L1 and IDOi, but no further improvement of tumor control was observed (data not shown). To investigate the interplay between inhibitory receptor blockade and IDO expression, we treated tumor-bearing mice with αPD-L1 or IDOi alone and harvested the tumor on the last day of therapy for analysis of IDO1 and PD-L1 transcripts by qRT-PCR. While PD-L1 mRNA expression was not altered by either treatment, IDO1 mRNA expression was enhanced with either treatment (Additional file 2: Figure S1). These results indicate that immune responses augmented with αPD-L1 may secondarily lead to further upregulation of IDO, and provide an additional rationale for combination therapy.

To assess if the anti-tumor effect was restricted to the presence of the model-antigen SIY we also evaluated all three double therapies using the parental cell line B16F10 (Additional file 2: Figure S2). Using the same dose of tumor cells as for B16.SIY we detected a significant delay in tumor outgrowth for all three double therapies compared to the no treatment control, although the effect was less dramatic with this less immunogenic variant. To investigate whether the doublet therapy would also mediated tumor control in larger more established tumors, we delayed the initiation of treatment until day 7. As depicted in Additional file 2: Figure S3, delayed treatment with αCTLA-4 + αPD-L1 also resulted in significant tumor control compared to the no treatment, although therapeutic efficacy was less potent than with earlier treatments. Together, these results suggest that combinatorial targeting of CTLA-4 +/− PD-L1 +/− IDO could translate into a therapeutic advantage in vivo.

### Effective combination therapies do not substantially increase the frequency of anti-tumor CD8^+^ T cells in the tumor-draining lymph node at early time points

**Figure 2:**
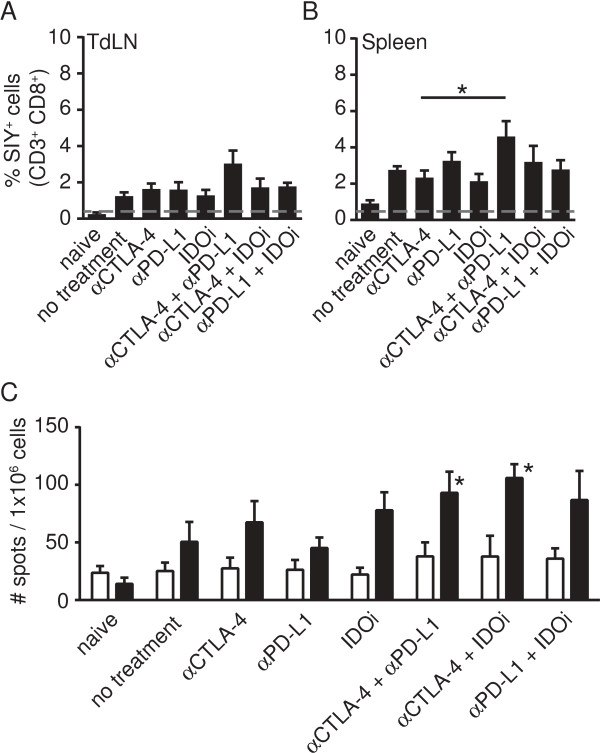
**Double regimen therapy does not result in substantially increased frequency of tumor-reactive T cells at early time points in the periphery. A**-**B**. Peptide/K^b^ pentamer staining was performed on gated CD3^+^CD8^+^ T cells, isolated from TdLN **(A)** or spleen **(B)** on day 7. Shown are means +/− SEM of a total of 10 mice collected from two experiments. For statistical analysis, Mann–Whitney-U test was performed comparing single treatments to double treatments (* = 0.0317 αCTLA-4 to αCTLA-4 + αPD-L1 in spleen). None of the other treatments was significantly different to the no treatment control. **C**. IFN-γ ELISpot was performed on splenocytes collected on day 7. Data are shown as mean +/− SEM from 10 mice out of two experiments with no stimulation as open bar and SIY-specific stimulation as filled bar. The Mann–Whitney-U test was used to assess significant differences between the no treatment group and treatment regimens with * in the figure indicating significant difference compared to no treatment. Results with αCTLA-4 were significantly different to αCTLA-4 + IDOi (p = 0.0185) and αPD-L1 was significantly different to αCTLA-4 + αPD-L1 (p = 0.0172).

We then turned toward understanding the mechanism by which improved immune-mediated tumor control might have been achieved. The major distinction was to assess whether successful doublet therapies were first improving the de novo priming of anti-tumor T cells in the TdLN which then subsequently homed to tumor sites for improved tumor control, versus directly restoring or augmenting the function of CD8^+^ T cells already infiltrating the tumor microenvironment. To test the first hypothesis, we assessed the frequency of SIY-specific CD8^+^ T cells in the TdLN and in the spleen on day 7, using SIY-K^b^ pentamer staining (Figure 2A, B). However, only minimal increases in frequencies of tumor-specific CD8^+^T cells were observed. The functional capacity of these cells was further assessed by IFN-γ ELISpot (Figure 1C). Using this assay as well, we failed to detect major differences between treatment groups. Only αCTLA-4 + αPD-L1 and αCTLA-4 + IDOi showed a statistically significant difference compared to no treatment (p = 0.0263 and p = 0.0101) as well as to their respective single treatments (αCTLA-4 + αPD-L to αPD-L1 p = 0.0172; αCTLA-4 + IDOi to αCTLA-4 p = 0.0185). However, this difference did not seem sufficient to account for the major improvement in tumor control observed.

Recent studies have focused on the potential Treg-depleting effect of αCTLA-4 antibody therapy [36]. To address this possibility in our model, we evaluated Treg frequencies using FoxP3 intracellular flow cytometry analysis in the lymphoid organs and within the tumor. However, neither on day 7 nor on day 14 were we able to detect a decrease in Treg frequency in any organ (Additional file 2: Figure S4, Additional file 1: Table S2). This difference might be explained by different αCTLA-4 mAb being used in various studies, and exclude a requirement for Treg depletion in the efficacy of these combination therapies in our model system.

### Effective doublets result in increased frequency of IL-2-producing and proliferating polyfunctional T cells within the tumor

**Figure 3:**
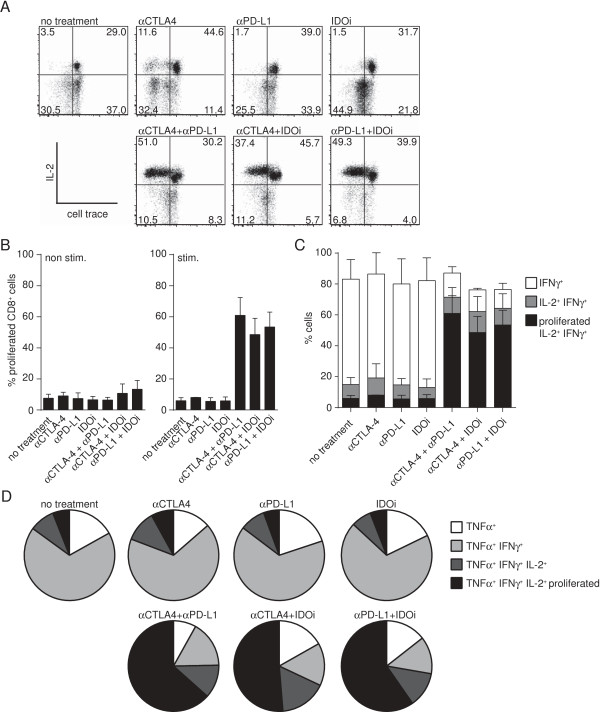
**Double treatments restore capacity of lymphocytes within the tumor to produce IL-2 and proliferate.** Tumors were harvested on day 7 and single cell suspensions were prepared. Pools of cells from 3–5 mice were combined and subsequently stained with cell trace. Cells were cultured with or without plate-bound anti-CD3 for 48 h then treated with anti-CD3/anti-CD28-stimulation in the presence of Brefeldin A for 6 h. Cells were then stained for production of IL-2, IFN-γ and TNF-α by intracellular flow cytometry. **A**. A representative FACS plot showing proliferation via cell trace dilution on the x-axis and intracellular IL-2 staining on the y-axis. A pool of five mice was analyzed. **B**. Statistical analysis of the amount of proliferating CD3^+^CD8^+^ cells in the non-stimulated (left) and stimulated (right) group. Only double treatments show a significant increase in proliferation compared to non-stimulated and single treatments when tested with a one-way Anova. Bars represent mean +/− SEM of a total of 4 pools collected out of 2 experiments. **C**. The percentages of IFN-γ^+^ (open bar), IFN-γ^+^ and IL-2^+^ (gray bar), and proliferating IFN-γ^+^IL-2^+^ cells (filled bar) were calculated within the CD3^+^CD8^+^ cell population. **D**. The percentages of polyfunctional (TNF-α^+^IFN-γ^+^IL-2^+^ proliferated) CD8^+^ T cells (black) compared to other less functional subsets is shown. Dark gray represents no proliferation, light gray represents no IL-2 production, white represents no IFN-γ production for each treatment group. **C** and **D** were calculated based on the results shown in **A** and **B**.

In order to address the second hypothesis of whether the therapeutic effect was a result of reactivation of tumor-infiltrating CD8^+^ T cells, we utilized an ex vivo stimulation protocol which is designed to favor analysis of pre-activated T cells. Responsiveness was assessed by measuring proliferation (CellTrace dilution) as well as production of IL-2, IFN-γ and TNF-α (tumor necrosis factor alpha) by intracellular cytokine staining. As depicted in a representative flow cytometric plot in Figure 3A, only the therapeutic effective doublet treatments showed a detectable proliferation rate of CD8^+^ T cells in combination with IL-2 production, and the magnitude of this effect was striking (non-stimulated control shown in Additional file 2: Figure S5). A statistical analysis of data spanning two independent experiments confirmed that significant ex vivo proliferation was only observed in stimulated T cells from mice that received the effective doublets (Figure 3B). Modest increases in both IFN-γ and TNF-α production were also seen; however, high production of these cytokines by tumor-infiltrating CD8^+^ T cells was already observed without treatment. Looking further at IFN-γ-producing cells in the doublet treatment groups, we observed most IFN-γ-producing T cells were also positive for IL-2 production and showed significantly increased proliferation (Figure 3C), while single treatment groups showed mainly IFN-γ single-producing cells. Comparing the stimulated cells to non-stimulated controls confirmed that only the double treatments were able to show increased IL-2 production and proliferation above background levels (Additional file 2: Figure S5). These T cells also produced TNF-α, indicating a polyfunctional T cell phenotype (Figure 3D) [37-39]. Detection of cytokine production was dependent on re-stimulation in vitro (Additional file 2: Figure S5). Thus, all three effective immunotherapy doublets resulted in the same improved functional effect: restoration of IL-2 production and proliferation by CD8^+^ intratumoral T cells, along with augmented polyfunctionality.

### Increased frequency of polyfunctional T cells within the tumor does not require new T cell migration

**Figure 4:**
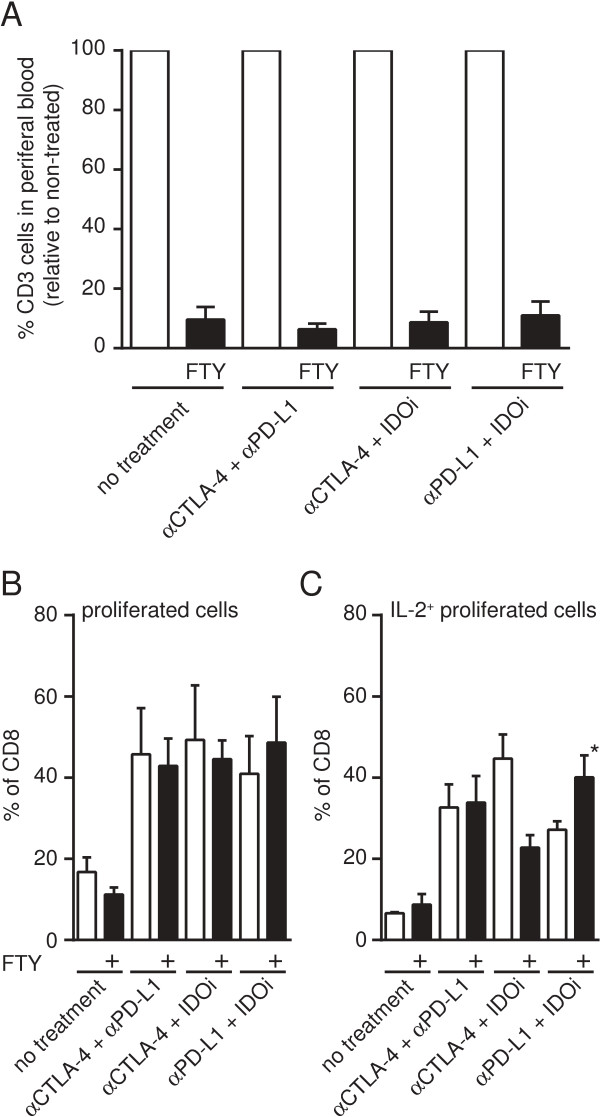
**Restoration of IL-2 production and proliferation of tumor-infiltrating lymphocytes in the absence of new T cell migration.** B16.SIY-bearing mice were either treated with FTY720 or control vehicle prior to initiation of therapy, to prevent migration of new lymphocytes into the tumor. **A**. Peripheral blood T cell numbers following FTY720 treatment on the day of tumor harvest for analysis. Open bars depict the number of CD45^+^CD3^+^ T cells detected in 200ul peripheral blood of vehicle treated mice set to 100%. Filled bars represent the number found in FTY720-treated mice, relative to the vehicle-treated group. **B** &**C**. Single cell suspensions from tumor were labeled with cell trace and stimulated with plate-bound anti-CD3 antibody for 48 h prior then with anti-CD3 and anti-CD28 in the presence of Brefeldin A. Cells were then analyzed for proliferation by cell trace dilution and production of IL-2 via intracellular staining. Depicted are the percentages of proliferating cells **(B)** or proliferating and IL-2 producing cells **(C)** comparing vehicle-treated groups (open bar) to FTY720-treated groups (filled bar). Results are shown as the mean +/− SEM combining two experiments with each having 2 pools of 3 mice. Significance was tested using Mann–Whitney-U test but no significant change between FTY720 and vehicle control could be detected except for increased IL-2 production in the αPD-L1 + IDOi treatment group (p = 0.02).

It was of interest to determine whether the presence of CD8^+^ T cells showing high levels of IL-2 production and proliferation within the tumor microenvironment following effective immunotherapy doublets was a result of new T cell migration into the tumor site versus re-activation of T cells already present. To test this hypothesis, we utilized FTY720 treatment to block the sphingosine 1-phosphate receptor-1 and thereby prohibit T cells from exiting lymphoid organs. Previous studies have shown that the effect can be detected as soon as 2 h after initial administration and is stable for up to 4 days [40]. We therefore administered FTY720 or control vehicle to tumor-bearing mice on day 4, 2.5 h prior to the initiation of immunotherapies. To control for effective depletion of circulating T cells we assessed the number of CD3^+^ cells in the peripheral blood. Overall, we detected a 90% reduction in circulating CD3^+^ T cells in FTY720-treated mice (Figure 4A). We then analyzed whether IL-2 production and proliferation by intratumoral CD8^+^ T cells were still restored. In fact, comparing vehicle-treated groups (open bars) to the FTY720-treated groups (filled bars) a similar increase in proliferation and IL-2 production was observed (Figure 4B, C), although there were some subtle differences. A modest reduction of proliferated/IL-2^+^-cells could be detected after αCTLA-4 + IDOi treatment and a modest increase could be observed when mice were treated with αPD-L1 + IDOi (p = 0.0286) (Figure 4C). Nevertheless, these data strongly suggest that the major mechanism for improved T cell function within the tumor microenvironment following these effective immunotherapies is through a direct effect on CD8^+^ T cells already present in the tumor site.

**Figure 5:**
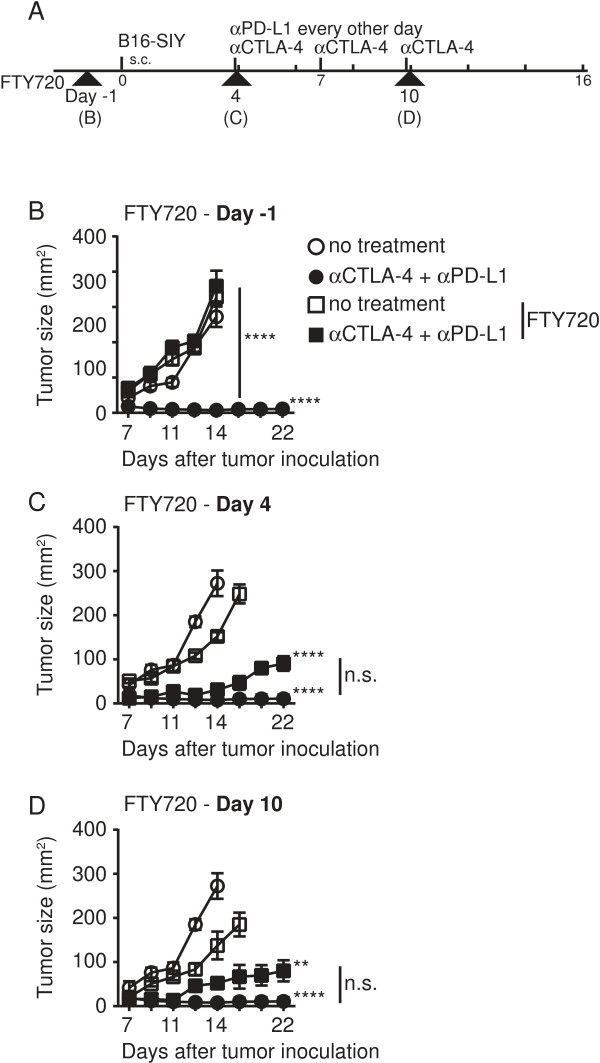
**Tumor control is largely preserved despite FTY720 administration at later time points.** B16.SIY-bearing mice were either treated with FTY720 or control vehicle prior to tumor inoculation **(B)**, prior to initiation of therapy **(C)** or with the last dose of αCTLA-4 mAb on day 10 **(D)**. **A**. Schematic overview of the experimental design bold arrow heads indicate initiation of FTY720 treatment, which was always carried out through the entire experiment. **B**-**D**. Tumor outgrowth measured in mm^2^ comparing vehicle control (circle) to FTY720 treatment (square) on no treatment (open) vs. αCTLA-4 + αPD-L1 (filled). **B**. FTY720 treatment was initiated 24 h prior to tumor inoculation. **C**. FTY720 treatment was given on day 4, 2.5 h before therapy was initiated. **D**. FTY720 was given at the same time point as the last dose of αCTLA-4 mAb treatment. Depicted is a representative experiment, showing mean of 5 mice +/− SEM. Significance was determined using a two-way ANOVA with Bonferroni post-test.

In addition to effects on TIL function, we further investigated if tumor regression induced by treatment doublets also could be preserved despite FTY720 administration. To test this notion we gave FTY720 over the course of the experiment starting at various time points (illustrated in Figure 5A). Administration of FTY720 on day −1, prior to tumor inoculation, abolished any therapeutic effect by αCTLA-4 and αPD-L1 (Figure 5B). However, we surprisingly observed that FTY720 treatment on day 4 or day 10 (after tumors were established, TILs were present, and therapy had been initiated) only a minimal loss of tumor control occurred (Figure 5C and 5D). These data support the notion that the majority of tumor control induced by αCTLA-4 and αPD-L1 is mediated by T cells that have already left secondary lymphoid organs and presumably reside already in the tumor site.

**Figure 6:**
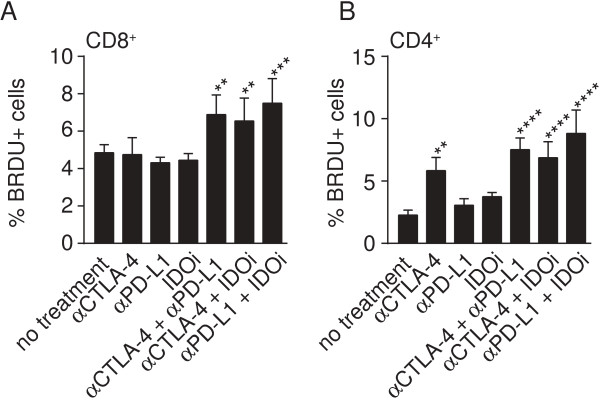
**Immunotherapy doublets result in increased BrdU incorporation by CD8**^**+ **^**and CD4**^**+ **^**tumor-infiltrating T cells *in vivo*.** TILs were harvested on day 7, 24 h after a single BrdU pulse in vivo, and cells were stained for BrdU along with anti-CD3, anti-CD4, and anti-CD8. Depicted are percentages of BrdU^+^ cells that were CD3^+^ CD8^+^**(A)** and CD3^+^ CD4^+^**(B)**. Data shown present the mean of a total of 5 mice from one experiment and are representative of two independent experiments. Differences were assessed using a two-way Anova test and taking proliferation values from spleen and TdLN into account. * indicates significantly different to no treatment and all double treatments were significantly different to their corresponding single treatments with the exception of αCTLA-4 to both double treatments for CD4 T cell proliferation.

To further address whether increased proliferation of tumor-infiltrating T cells was induced upon treatment with effective immunotherapy doublets, we utilized a short pulse of BrdU (Bromodeoxyuridine) administration in vivo. To this end, a single dose of BrdU was administered i.p. on day 6 and proliferation of T cells in the tumor (Figure 6), spleen and TdLN (Additional file 2: Figure S6) was assessed on day 7. Consistent with our ex vivo results, we observed that the doublet-treated mice harbored more proliferating CD8^+^ T cells in the tumor site than did the single-treated groups or the mice that received no treatment (Figure 6A). A similar effect was also seen for CD4^+^ T cells, with the exception that αCTLA4mAb single treatment also resulted in an increased proliferation rate in this cell compartment (Figure 6B). Taken together, these data indicate that the therapeutically successful combination therapies of αCTLA-4 +/− αPD-L1 +/− an IDOi resulted in increased proliferation and reactivation of CD8^+^ T cells directly within the tumor site.

### Combinatorial treatments lead to prolonged persistence and higher frequency of tumor-reactive lymphocytes in the periphery at later time points

**Figure 7:**
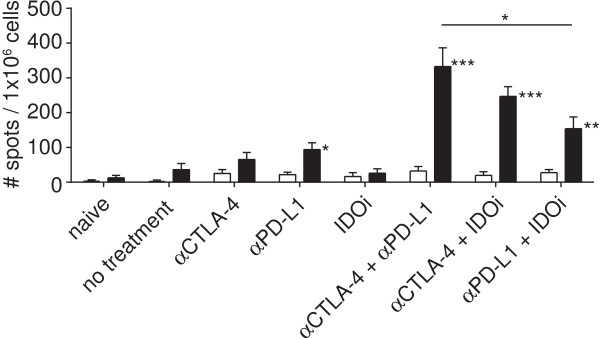
**Immunotherapy doublets result in increased frequencies of tumor antigen-specific T cells at later time points in the periphery.** Depicted is an IFN-γ ELISpot of splenocytes harvested on day 14 with open bars being the un-stimulated control and filled bars representing SIY-stimulation. Data are shown as the mean +/− SEM from 10 mice pooled from two experiments. Statistical analysis was done using Mann–Whitney-U test comparing all groups to no treatment. All double treatment groups were significantly different to their respective single treatment and when comparing double treatments within each other a significant difference between αCTLA4 + αPD-L1 and αPD-L1 + IDOi was observed.

Effective non-specific immunotherapies are often suggested to result in increased tumor antigen-specific T cell frequencies in peripheral lymphoid organs, yet our results above have indicated that this was not the case at early time points in this model. We reasoned that, if these treatments are indeed restoring T cell function within the tumor site, then once tumors start to regress after therapy, some of these anti-tumor T cells may recirculate and become detectable in the periphery. To test this notion we assayed for functional SIY-specific T cells in the spleen on day 14 (Figure 7). Indeed, a significant increase in the frequency of IFN-γ-producing T cells upon SIY stimulation was observed when comparing the double treatments to the single treatment or no treatment groups. All three of the double treatment groups show an increase in SIY-reactive cells by 2- to even 3.5-fold higher levels compared to day 7. This effect was accompanied by increased frequency of SIY-pentamer positive CD8^+^ T cells in the spleen, and also within the tumor (Additional file 2: Figure S7). Consistent with these results, re-challenge of mice 6 weeks later that rejected the first tumor were protected against B16.SIY (Additional file 1: Table S1). Thus, the successful doublet treatments eventually led to a higher circulating fraction of tumor antigen-specific T cells that likely represents a memory response.

## Discussion

Our current work has indicated that doublet therapies using either αCTLA-4, αPD-L1 and/or IDOi show a synergistic retardation of tumor outgrowth in vivo. The major biologic correlate to this improved efficacy was restored IL-2 production and proliferation of tumor-infiltrating CD8+ T cells. In addition, this functional restoration did not require new T cell migration as assessed using FTY720 administration. Together, these data suggest that successful combination immunotherapies function, at least in part, by correcting functional defects of T cells directly within the tumor microenvironment.

It is noteworthy that CD8^+^ TILs (tumor-infiltrating lymphocytes) without any therapy showed significant production of IFN-γ production when analyzed ex vivo. Consistent with this observation, human melanoma metastases showing a T cell-infiltrated phenotype usually show expression of IFN-γ-induced target genes and in many cases IFN-γ itself [41]. Recently, we have observed that IFN-γ produced by CD8^+^ T cells is necessary for the induction of the negative regulatory factors PD-L1 and IDO within the tumor microenvironment [12]. Our current data indicate that IDO1 is further upregulated upon blockade of PD-L1/PD-1 interactions. Thus, the retained ability of TIL to produce at least some IFN-γ may in fact contribute to the negative regulatory network within the tumor site that enable tumor immune evasion.

Although several previous studies have been conducted to investigate if combining blockade of two inhibitory pathways could act synergistically in retarding the tumor growth in vivo, only limited studies have addressed the mechanism leading to this effect [30,33,42,43]. In particular the recently published studies by Duraiswamy and colleagues (αCTLA-4 + αPD-L1) and Holmgaard et al. (αCTLA-4 + IDOi) indicated that proliferation of TIL in combination with increased functional capacity (IFN-γ, granzyme B) were increased with effective combinations [44,45]. Taken together, these collective results converge on the notion that restored function of existing TIL may be a critical mechanism of action of these interventions. Presence of polyfunctional T cells has been described to be associated with improved anti-tumor immunity in preclinical mouse models and also in patients [37,38,46]. It is not yet known if other immunotherapy doublets will similarly involve reactivation of T cells only within the tumor microenvironment. Nonetheless, the data presented here show a striking importance of TIL reactivation mediated by the doublet therapies we have tested. The mechanism leading to this could be compensatory immune regulation by a second pathway when only one pathway is inhibited.

Recent studies have suggested that αCTLA-4 antibody could lead to depletion of regulatory T cells selectively within the tumor microenvironment [36,47]. In our own hands, we were not able to detect diminished Tregs after αCTLA4 mAb either alone or in combination, either measuring percentage or absolute number in the tumor site. The reason for this discrepancy is not clear, but could be related to the use of distinct mAbs against CTLA-4. While studies by Allison et al. were performed with the 9D9 mAb (mouse IgG2b), our study was conducted with the 4 F10 mAb (hamster IgG1). Further work will be required to determine under what circumstances Treg depletion may be functionally relevant with αCTLA-4 agents. Our own data suggest that it is not mandatory for αCTLA-4 to deplete intratumoral Tregs in order to have therapeutic benefit in vivo.

Our experiments with FTY720 suggest that new T cell migration is not required in order to improve CD8^+^ T cell function in response to immunotherapy combinations in vivo. However, it is clear that successful immunotherapies trigger tumor cell death, renewed inflammation in the tumor site, antigen cross-presentation, and epitope spreading [48], ultimately leading to increased T cell infiltration in the tumor microenvironment. This entire cascade of events may be required for optimal tumor elimination. Indeed, our experiments of FTY720 administration at later time points showed a modest effect on tumor growth control. However, our results demonstrate that new T cell migration is not required for the early improved TIL function observed as well as for maintaining a steady-state control of the tumor outgrowth. These data have limitations, in that approximately 10% of peripheral blood T cells were still present after FTY720 treatment, which could potentially contribute to tumor infiltration. However, an important contribution of new migration in the presence of FTY720 would likely have been associated with a similar 90% reduction of T cell numbers in the tumor which is indicated by the blunted tumor control when FTY720 is administered on day −1. In addition, our direct in vivo analysis of TIL proliferation using BrdU administration support the notion of rapid restoration of TIL proliferation in situ.

## Conclusions

In total, the results presented herein suggest that doublet combinations of anti-CTLA-4, anti-PD-1/PD-L1, or IDO inhibition are attractive for clinical translation. Phase I clinical trial development of the anti-CTLA-4 + anti-PD-1 combination has already been pursued in advanced melanoma, with profound clinical response rates observed that appear superior to single agent activity based on historical controls [47,49]. Development of the other combinations described herein may have a similarly strong rationale for clinical testing. Our data also suggest that proliferation and/or IL-2 production by CD8^+^ TIL should be considered as a pharmacodynamic biomarker for clinical response to these combinations.

## Methods

### Mice and tumor inoculation

C57BL/6 mice were purchased from Taconic Farms and were maintained according to National Institute of Health Animal Care guidelines. Ethical approval was obtained by the Council for Animal Research at The University of Chicago and followed international guidelines. B16F10 and B16-dsRed-SIY tumor cells [50] were maintained as previously described and B16-dsRed-SIY will be specified as B16.SIY throughout the paper. On day 0 of the experiments, 2×10^6^ B16-dsRed-SIY cells were inoculated subcutaneously into the flank of the mice. Mice with same age and gender were used as controls.

### Treatment regimens

Treatment was initiated on day 4 post tumor inoculation with the following regimens for each drug (illustrated in Figure 1A). αCTLA-4 antibody (clone UC10-4 F10-11, Bio-X-Cell) was given i.p. on day 4, 7 and 10 at a dose of 100 μg/mouse. αPD-L1 antibody (clone 10 F.9G2, Bio-X-Cell) was given i.p. (100 μg/mouse) every other day starting on day 4 ending on day 16 post tumor inoculation. IDOi (INCB23843, Incyte Corporation) was dissolved in 0.5% methylcellulose and administered at 300 mg/kg po QD on a 5 days on/2 days off schedule starting on day 4 [35,51]. In the case of functional experiments with an earlier endpoint, treatment regimens were carried out as described until the day of T cell analysis. For delayed therapy the same treatment scheme was applied starting on day 7 post-tumor inoculation, when tumors were palpable.

### Flow cytometry and antibodies

For flow cytometric analysis, spleen, TdLN, and tumor tissues were harvested at the indicated time point or when tumors reached a volume of 200 mm^2^. Single cell suspensions were prepared and a Ficoll-Hypaque purification step was performed for the tumor-derived cell suspension. Following a washing step, approximately 2×10^6^ cells were used for antibody staining. Antibodies against the following molecules were used throughout the paper if not otherwise indicated: CD3 (AX700, 17A2, eBioscience), CD4 (PerCP-Cy5.5, RM4-5, Biolegend), CD8 (APCCy7, 53–6.7, Biolegend), FoxP3 (APC, FJK-16a, eBioscience), IL-2 (PerCP, JES6-5H4, eBioscience), IFN-γ (APC, XMG1.2, eBioscience), and TNF-α (FITC, MP6-XT22, eBioscience). Fixable life/dead cell discrimination was performed using Fixable Viability Dye 450 or 506 (eBioscience). Staining was carried out at RT for 30 min if not indicated differently and intracellular staining was performed using the FoxP3-staining kit according to manufacturer’s instructions (BD).

Staining of SIY-specific cells was performed using the SIYRYYGL-pentamer (Proimmune), conjugated with Phycoerythrin (PE), or as a non-specific control with the SIINFEKL-pentamer. For staining, pentamers were diluted 1:50 in PBS + 10% FCS and incubated for 20 minutes at room temperature (RT). Following a washing step, cells were stained with specific antibodies for 30 minutes on ice prior to fixation in 4% PFA. All flow cytometric analyses were done using an LSR II blue instrument (BD) and analyzed using FlowJo software (Tree Star).

### IFN-γ ELISpot

Splenocytes from naïve, tumor-challenged non-treated or treated mice were harvested on day 7 or day 14 after tumor inoculation. Single cell suspensions were prepared and 1×10^6^ splenocytes were assayed per well. Cells were either left un-stimulated or stimulated with 160nM SIY-peptide (SIYRYYGL) or PMA 100 ng/ml and Ionomycin 1 μg/ml as positive control. After a 24 h culture period, detection of INF-γ production was performed according to manufacturer’s instructions.

### Quantitative RT-PCR analysis

Tumor was harvested on day 16 and a single cell suspension was prepared. Approximately 10^7^ cells were used for RNA isolation using Qiagen RNAeasy extraction kit according to manufactured instruction. Following cDNA was prepared using Reverse Transcriptase kit (Manufactures instructions; Applied Biosciences). Expression levels of transcripts were analyzed using primer-probe sets specific for IDO1 and PD-L1 and values were normalized against the expression level of 18S (Roche).

### Ex vivo T cell functional assays

Single cell suspensions from tumor, spleen, and TdLN were prepared as described above. Cell numbers were determined and cells were labeled with Cell Trace (BD) according to manufacturer’s instructions. A maximum of 1×10^6^ cells was plated per well on either non-treated or anti-CD3 mAb-coated plates. Anti-CD3 mAb coating was performed with a solution of 10 μg/ml αCD3 antibody (145-2C11, Biolegend) in PBS, incubated overnight at 4°C. Following 48 h of incubation, cells were harvested and transferred onto newly anti-CD3-coated or non-treated plates, along with anti-CD28 mAb (2 μg/ml) (EL-4, Biolegend). Medium for all wells included 5 μg/ml BrefeldinA (Sigma). Following a 6 h-incubation at 37°C, cells were harvested and stained for surface markers and intracellular cytokines using the technique described above.

### Treatment with FTY720

Prior to the initiation of the therapy regimens (2.5 h pre-treatment), fingolimod (FTY720, Enzo Life Sciences) was given to mice to inhibit lymphocyte migration out of lymphoid organs [40]. FTY720 stock solution (10mg/ml in DMSO) was diluted to a 125μg/ml concentration in PBS directly before administration. Mice received a dose of 25μg FTY720 or PBS containing DMSO as control via oral gavage. Therapy was initiated the same day (2.5h delayed) and mice were analyzed on day 7 to perform the ex vivo functional assay as described above. For long-term FTY720 administration, the dose was reduced to 5μg per mouse per day and was given daily throughout the experiment (day-1 FTY720 was given 24h prior to tumor inoculation; day 4 FTY720 was given 2.5h prior to therapy; day 10 FTY720 was given 2.5h after last dose of αCTLA-4 mAb). Depletion of peripheral lymphocytes was assessed on the endpoint of each group and was always detected to be greater than 90% depletion.

### In vivo proliferation assay

Assessment of in vivo proliferation was performed by BrdU pulse in vivo, 24 h prior to flow cytometric analysis. Each mouse received 0.8 mg BrdU in 100 μl injected i.p. either on day 6 or day 13 of the treatment protocol. Mice were analyzed on day 7 or day 14, respectively, and cells were prepared for flow cytometry as described above. Following surface staining, cells were fixed using the FoxP3 staining kit (BD). After the 30 minute fixation period, cells were incubated in 100 μl of PBS/DNase solution (300 μg/ml) for 30 minutes at 37°C. Cells were then washed and incubated for 30 minutes at RT with antibodies for FoxP3 and BrdU (FITC, Bu20a, eBioscience) followed by flow cytometric analysis.

## Abbreviations

APC: Allophycocyanin; AX700: AlexaFluor 700; BrdU: Bromodeoxyuridine; CTLA-4: Cytotoxic T-Lymphocyte Antigen-4; FITC: Fluorescein isothiocyanate; IDO: Indoleamine-2,3-dioxygenase; IDOi: IDO inhibitor; IFN: Interferon; IL: Interleukin; PD-L1: Programmed death-ligand 1; PD-1: Programmed death-receptor 1; PE: Phycoerythrin; PerCP: Peridinin chlorophyll protein complex; TdLN: Tumor-draining lymph node; TIL: Tumor-infiltrating lymphocyte; TNF-α: Tumor necrosis factor alpha; Tregs: Regulatory T cells.

## Competing interests

SS, BH and TG declare that they have no competing interests. HK, PS, RN are employees of Incyte Cooperation.

## Authors’ contributions

SS designed and performed all experiments and wrote the manuscript, TG designed the overall study design and contributed to writing and revising the manuscript. BH designed and performed experiments on delayed therapy. HK, PS, RN assisted with the study design and data interpretation. All authors read and approved the final manuscript.

## Supplementary Material

Additional file 1: Table S1 Rechallenge of complete responders after therapy. **Table S2.** Percentages of FoxP3^+^ T cells (Tregs) in CD4 T cell population at day 7 and day 14 of the treatment regimen. Click here for file

Additional file 2: Figure S1 Expression of IDO1 and PD-L1 after therapy with IDO inhibitor or α PD-L1. **Figure S2.** Therapeutic doublets delay tumor outgrowth of B16F10. **Figure S3.** Therapy beginning later at day 7 also controls tumor outgrowth. **Figure S4.** Percentages of FoxP3^+^ T cells (Tregs) in CD4 T cell population at day 7 of the treatment regimen. **Figure S5.** Controls for ex vivo T cell functional assay. **Figure S6.** In vivo proliferation of CD8^+^ and CD4^+^ positive T cells in spleen and TdLN based on BrdU uptake. **Figure S7.** Immunotherapy doublets result in increased frequency and longer persistence of SIY/K^b^ pentamer-specific T cells in the periphery and in the tumor. Click here for file
