# Supplementary material for: Mechanism of tumor rejection with doublets of CTLA-4, PD-1/PD-L1, or IDO blockade involves restored IL-2 production and proliferation of CD8+ T cells directly within the tumor microenvironment
Source: J Immunother Cancer. 2014 Feb 18;2:3. doi: 10.1186/2051-1426-2-3 (PMC4019906; doi:10.1186/2051-1426-2-3)
Supplement: Additional file 1: Table S1 — Rechallenge of complete responders after therapy. Table S2. Percentages of FoxP3+ T cells (Tregs) in CD4 T cell population at day 7 and day 14 of the treatment regimen. [file 2051-1426-2-3-S1.docx]

**Table S1**: **Rechallenge of complete responders after therapy.**

Mice with a complete rejection of the tumor after therapy were rechallenged with 2x10^6^ B16-SIY cells 4 weeks after therapy was ended. Tumor growth was followed up to 8 weeks after rechallenge.

| **treatment** | **# mice** | **# complete rejection** | **# rechallenged** | **# protective response** |
| --- | --- | --- | --- | --- |
| αCTLA-4 | 37 | 0 | 0 | 0 |
| αPD-L1 | 31 | 0 | 0 | 0 |
| IDOi | 26 | 0 | 0 | 0 |
| αCTLA-4 + αPD-L1 | 27 | 15 | 5 | 4 |
| αCTLA-4 + IDOi | 16 | 3 | 3 | 2 |
| αPD-L1 + IDOi | 15 | 2 | 2 | 0 |

**Table S2**: **Percentages of FoxP3+ T cells (Tregs) in CD4 T cell population at day 7 and day 14 of the treatment regimen.**

Percent of FoxP3+ CD4 T cells with ± SEM (italic) on day 7 and day 14 post tumor inoculation with the indicated treatment regimens in Tumor draining lymph node (TdLN), spleen and tumor (n=10 pooled out of two independent experiments).

| ***Day7*** | naive | | no treatment | | αCTLA-4 | | αPD-L1 | | IDOi | | αCTLA-4 + αPD-L1 | | αCTLA-4 + IDOi | | αPD-L1 + IDOi | |
| --- | --- | --- | --- | --- | --- | --- | --- | --- | --- | --- | --- | --- | --- | --- | --- | --- |
| TdLN | 11.95 | *0.62* | 11.04 | *0.71* | 15.15 | *0.52* | 11.57 | *0.80* | 14.27 | *0.32* | 13.84 | *0.43* | 12.65 | *0.64* | 17.12 | *0.73* |
| Spleen | 12.65 | *0.35* | 14.43 | *0.92* | 15.53 | *0.95* | 18.23 | *1.15* | 19.50 | *1.73* | 19.55 | *0.76* | 17.03 | *0.54* | 22.12 | *1.33* |
| Tumor |  |  | 23.97 | *1.76* | 22.70 | *2.67* | 22.05 | *0.90* | 25.02 | *1.67* | 21.49 | *1.16* | 16.82 | *1.72* | 21.58 | *1.72* |
|  |  |  |  |  |  |  |  |  |  |  |  |  |  |  |  |  |
|  |  |  |  |  |  |  |  |  |  |  |  |  |  |  |  |  |
| ***Day14*** | naive | | no treatment | | αCTLA-4 | | αPD-L1 | | IDOi | | αCTLA-4 + αPD-L1 | | αCTLA-4 + IDOi | | αPD-L1 + IDOi | |
| TdLN | 9.90 | *0.45* | 10.94 | *0.19* | 15.12 | *1.50* | 12.74 | *0.57* | 11.20 | *0.38* | 14.72 | *0.50* | 12.40 | *0.64* | 14.02 | *0.74* |
| Spleen | 19.44 | *1.77* | 20.16 | *1.28* | 20.04 | *1.21* | 25.58 | *0.84* | 24.16 | *2.32* | 19.40 | *0.73* | 20.84 | *1.80* | 22.24 | *0.85* |
| Tumor |  |  | 26.52 | *2.08* | 23.56 | *5.83* | 26.08 | *2.11* | 24.40 | *2.63* | **11.77** | ***4.46*** | 17.88 | *1.23* | 35.22 | *9.17* |
