# Supplementary material for: Mechanism of tumor rejection with doublets of CTLA-4, PD-1/PD-L1, or IDO blockade involves restored IL-2 production and proliferation of CD8+ T cells directly within the tumor microenvironment
Source: J Immunother Cancer. 2014 Feb 18;2:3. doi: 10.1186/2051-1426-2-3 (PMC4019906; doi:10.1186/2051-1426-2-3)
Supplement: Additional file 2: Figure S1 — Expression of IDO1 and PD-L1 after therapy with IDO inhibitor or α PD-L1. Figure S2. Therapeutic doublets delay tumor outgrowth of B16F10. Figure S3. Therapy beginning later at day 7 also controls tumor outgrowth. Figure S4. Percentages of FoxP3+ T cells (Tregs) in CD4 T cell population at day 7 of the treatment regimen. Figure S5. Controls for ex vivo T cell functional assay. Figure S6. In vivo proliferation of CD8+ and CD4+ positive T cells in spleen and TdLN based on BrdU uptake. Figure S7. Immunotherapy doublets result in increased frequency and longer persistence of SIY/Kb pentamer-specific T cells in the periphery and in the tumor. [file 2051-1426-2-3-S2.docx]

**Supplementary Material**

**
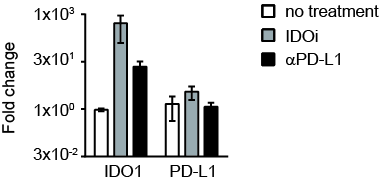
**

**Figure S1.** **Expression of IDO1 and PD-L1 after therapy with IDO inhibitor or αPD-L1.**

Mice were injected with 2x10^6^ B16-SIY tumor cells and monotherapy was initiated as described. On day 14, tumors were isolated and RNA was extracted from single cells suspensions. Specific transcript levels were assessed using qRT-PCR. Levels were normalized to the expression level of 18S and are depicted as relative fold change to non-treated mice (2^-(dCT_WT_-dCT_test_). Shown are means from 3 mice each +/- SEM (WT open, IDOi gray, αPD-L1 black).

**
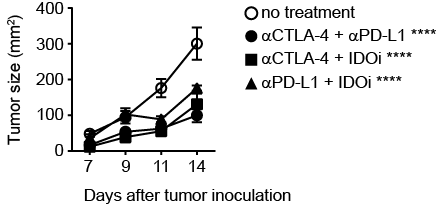
**

**Figure S2.** **Therapeutic doublets delay tumor outgrowth of B16F10.**

2x10^6^ B16F10 tumor cells were inoculated subcutaneously on day 0 and therapy was initiated on day 4 as described previously. Depicted are means from 5 mice +/- SEM, two-way ANOVA with Bonferroni post-test with **** being p<0.0001.

**
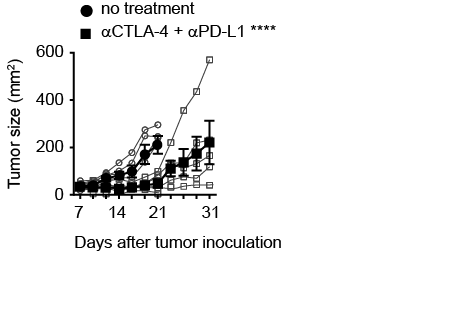
**

**Figure S3.** **Therapy beginning later at day 7 also controls tumor outgrowth.**

2x10^6^ B16-SIY tumor cells were inoculated subcutaneously on day 0 and therapy was initiated on day 7 when tumors were palpable. Depicted are means from 5 mice +/- SEM, two-way ANOVA with Bonferroni post-test with **** being p<0.0001. Additionally, gray symbols and lines show individual mice and indicated that major tumor control could be achieved in some but not all mice.


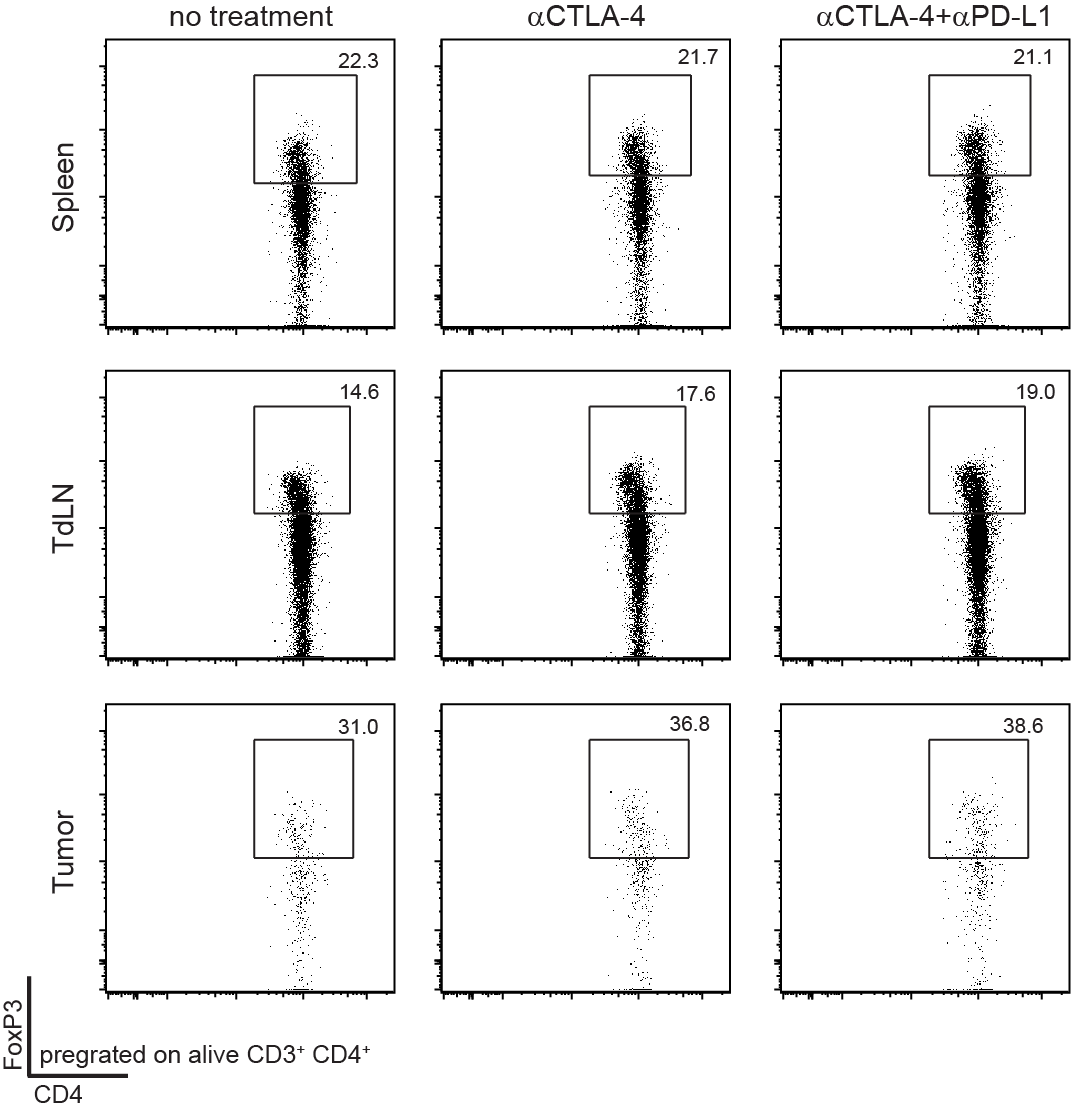


**Figure S4**: **Percentages of FoxP3+ T cells (Tregs) in CD4 T cell population at day 7 of the treatment regimen.**

Representative examples of FoxP3 flow cytometry staining blots, obtained on day 7 after tumor inoculation. Shown are examples for no treatment, αCTLA-4 and αCTLA-4+αPD-L1 treatment groups from spleen, tumor-draining lymph node (TdLN) and tumor. For statistical analysis see Table S2.

**
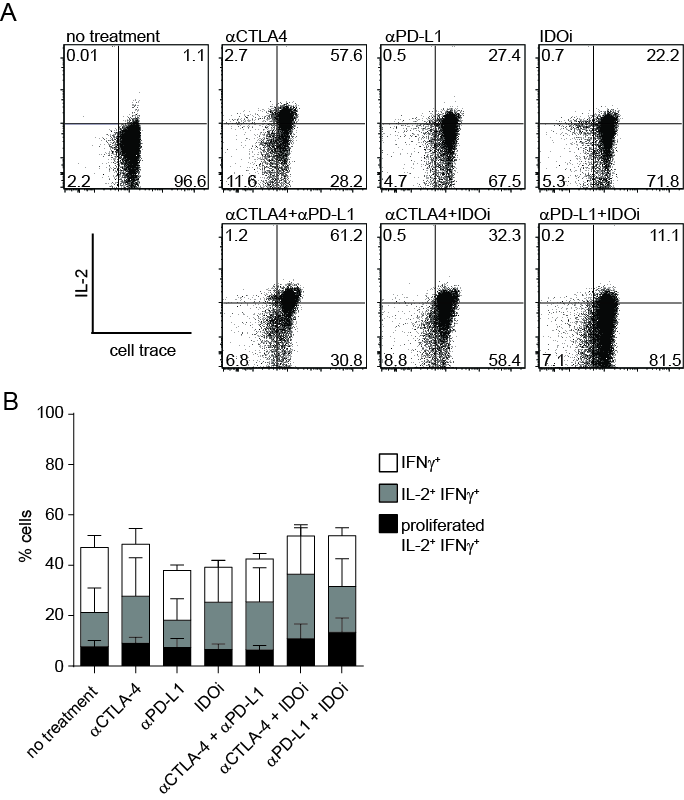
**

**Figure S5. Controls for ex vivo T cell functional assay.**

Depicted are the non-stimulated controls corresponding to Figure 3 A and C.


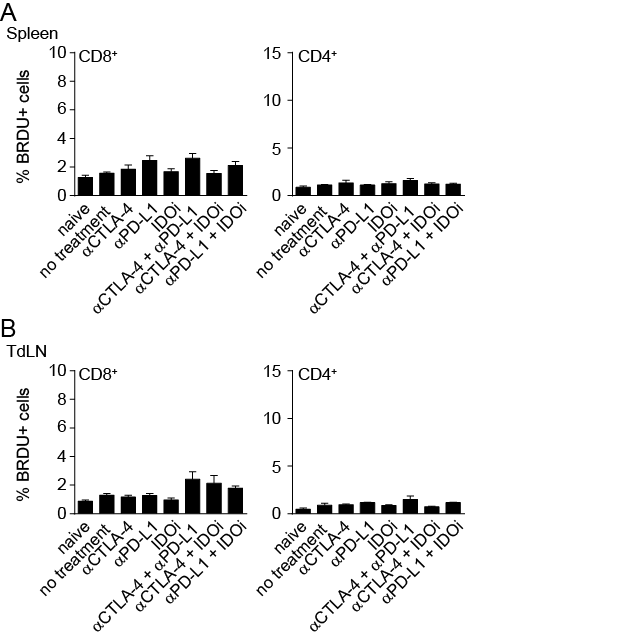


**Figure S6. In vivo proliferation of CD8+ and CD4+ positive T cells in spleen and TdLN based on BrDU uptake.**

BrdU staining was performed on cells from spleen and TdLN and analyzed side by side with the staining shown in Figure 5. Bars represent the mean +/- SEM out of a total of 10 mice. No differences were significant.


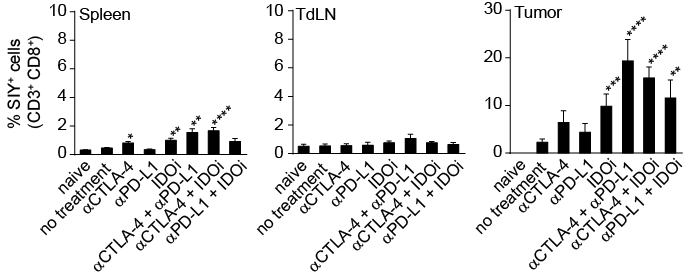


**Figure S7.** **Immunotherapy doublets result in increased frequency and longer persistence of SIY/K^b^ pentamer-specific T cells in the periphery and in the tumor.**

Pentamer staining was performed on gated CD3^+^CD8^+^ T cells, isolated from spleen, TdLN or tumor on day 14. Shown are means +/- SEM of a total of 10 mice collected from two experiments. For statistical analysis, Mann-Whitney-U test was performed comparing all treatments to the no treatment control (indicated by *). The following combinations were significantly different in the spleen: αCTLA4 to αCTLA4+αPD-L1 and αCTLA4+IDOi; αPD-L1 to αCTLA4+αPD-L1 and αPD-L1+IDOi; IDOi to αCTLA4+IDOi; and in the tumor: αCTLA4 to αCTLA4+αPD-L1 and αCTLA4+IDOi; αPD-L1 to αCTLA4+αPD-L1; IDOi to αCTLA4+IDOi.
